# Tumor treating fields alter local coagulation dynamics in glioblastoma patients

**DOI:** 10.1016/j.neurot.2025.e00715

**Published:** 2025-08-29

**Authors:** Claudia Maletzki, Thomas Freitag, Adrian Hempelmann, Annabell Wolff, Thomas M. Freiman, Sae-Yeon Won, Dirk Koczan, Sina Sender, Pablo A. Valdes, Joshua D. Bernstock, Florian Gessler, Daniel Dubinski

**Affiliations:** a Department of Medicine, Clinic III – Hematology, Oncology, Palliative Medicine, Rostock University Medical Center, University of Rostock, 18057 Rostock, Germany; b Department of Neurosurgery, Rostock University Medical Center, University of Rostock, 18057 Rostock, Germany; c Institute of Immunology, Rostock University Medical Center, University of Rostock, 18057 Rostock, Germany; d Institute of Diagnostic and Interventional Radiology, Pediatric Radiology and Neuroradiology, University Medical Center Rostock, 18057 Rostock, Germany; e Department of Neurosurgery, University of Texas Medical Branch, Galveston, TX 77555, USA; f Department of Neurosurgery, Brigham and Women's Hospital, Harvard Medical School, Boston, MA 02115, USA

**Keywords:** Hemostasis, Hypercoagulation, Cell death, Tissue factor, Clot rigidity

## Abstract

Glioblastoma (GBM) is a highly aggressive brain tumor, associated with hypercoagulability and thrombosis. Tumor Treating Fields (TTFields), a non-invasive therapy that uses low-intensity, alternating electric fields to disrupt cancer cell division, prolongs survival when used concomitantly with radiochemotherapy. TTFields-treated patients often exhibit distinct recurrence patterns, suggesting a local interaction between TTFields and tumor-associated coagulation, underlying mechanisms remain unclear. This study examined coagulation profiles in TTFields-treated patients’ blood, tumor cells, and plasma-derived extracellular vesicles using molecular, hemostaseologic, and phenotypic analyses. Our findings revealed that short-term TTFields exposure significantly prolongs blood coagulation in GBM patients and healthy donors by altering tissue factor (TF) expression and disrupting the extrinsic coagulation pathway. TTFields reduced clot rigidity by decreasing Factor II/FXIII activity and platelet count, without impairing fibrinogen function. Patient-derived GBM cells exposed to TTFields exhibited increased TF abundance. RNA-based microarray analysis of GBM cells confirmed coagulation-related changes, including upregulation of platelet adhesion marker *ITGA2*, and downregulation of *THBS1*, a regulator of clotting, platelet aggregation, extracellular matrix remodeling, and tumor invasiveness. Additionally, *TXNIP*, a coagulation-modulating gene, was downregulated after TTFields exposure, indicating a link to immune regulation in the tumor microenvironment. In an allogeneic co-culture model of patient-derived GBM cells and peripheral blood, TTFields modulated coagulation and immune responses, likely by rebalancing pro- and anticoagulant factors in the tumor microenvironment, reducing the prothrombotic state, and altering inflammatory pathways. These findings provide insights into how TTFields influence coagulation and, eventually, immune regulation, offering strategies to optimize clinical decision-making and mitigating thromboembolic complications in GBM patients.

## Graphical abstract

**Figure:**
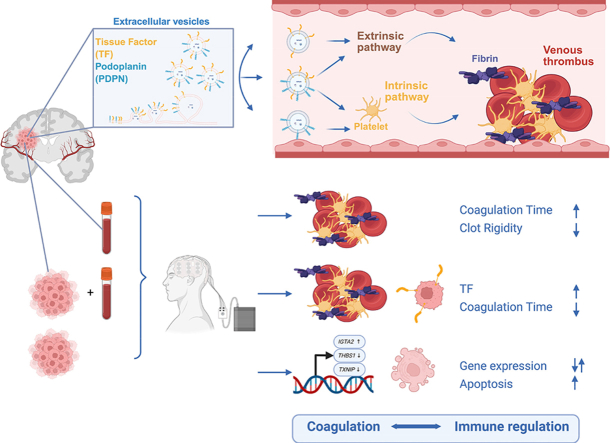

## Introduction

Glioblastoma (GBM), a primary WHO grade 4 brain tumor, is one of the most treatment-resistant human cancers, with an extremely poor overall survival rate [1,2]. The incidence of thrombosis in GBM patients is nearly double that of individuals with malignancies in other organ systems, with arterial and venous thromboembolism (VTE) posing a particularly severe complication during perioperative management [3,4]. VTE risk in GBM patients is up to 30 ​%, consistently remaining significantly higher throughout the disease course compared to other cancers [3,5].

The hypercoagulability observed in GBM arises from multiple factors, including vascular abnormalities, overexpression of tissue factor (TF) and podoplanin (PDPN), and the release of procoagulant extracellular vesicles (EVs) [6]. These EVs act as molecular mediators of both angiogenesis and thrombosis [7,8]. TF, a transmembrane glycoprotein overexpressed in GBM, initiates the clotting cascade by binding to Factor VII/FVIIa and is a central link between cancer and thrombosis [9]. Elevated local and circulating TF levels increase the risk of VTE in GBM, while lower-grade gliomas show reduced VTE risk due to isocitrate dehydrogenase-mutant (IDH^mut^) mediated gene methylation [10].

Tumor Treating Fields (TTFields) constitute a novel treatment modality for GBM, utilizing alternating electric fields with varying frequencies between 50 and 400 ​kHz to target tumor cells [11,12]. TTFields, when used alongside radiochemotherapy, have been shown to significantly improve overall survival in newly diagnosed and recurrent GBM by almost 4 months (20.9 vs 16.0 months) [12,13]. Preclinical studies revealed that TTFields induce cellular stress, autophagy, necroptosis, and immunogenic cell death with the release of HMGB1, ATP, and calreticulin translocation [14]. Ongoing clinical trials, including the EF-41/KEYNOTE D58 Phase 3 study (NCT06556563), are investigating the potential of TTFields-based therapies that extend beyond cytotoxicity to include immunomodulatory approaches. Before integrating novel combination therapies into clinical practice, a comprehensive understanding of the molecular impact of TTFields is essential, not only on malignant cells but also on their non-malignant counterparts. A key unresolved question remains how TTFields influence coagulation and their potential role in micro- and macrothrombosis in GBM patients, especially as these processes may indirectly impact immune responses.

In this study, we demonstrate, for the first time, that TTFields directly modulate coagulation by dysregulating TF activity and reprogramming thrombotic gene networks, while preserving fibrinogen function. Our findings suggest a link between coagulation and immune regulation in the tumor microenvironment, a relationship of considerable clinical significance that may guide the way to optimize anticoagulation strategies.

## Material & methods

### Ethical statement

All procedures involving patients were approved by the local Ethics Committee (Rostock University Medical Center, Ethics Registration ID: A2018-0167). All patients provided written informed consent to participate in the study.

### Cell culture

The patient-derived GBM cell lines: HROG04, HROG13, GBM06, and GBM15 were previously established and incubated in DMEM/Hams F12 medium, supplemented with 10 ​% FCS, 6 ​mM l-glutamine, and 1 ​% penicillin/streptomycin (all from PAN-Biotech, Aidenbach, Germany) and incubated at 37 ​°C in a humidified atmosphere of 5 ​% CO_2_. Continually growing cell cultures were serially passaged and regularly stored at low passages.

### Patient cohort and clinical characteristics

A total of 27 patients undergoing neurosurgical tumor resection at the University Medical Center Rostock were included in this study. Based on histopathological diagnosis, patients were grouped into four categories: glioblastoma (n ​= ​10), brain metastases (n ​= ​10), low-grade glioma (n ​= ​2), and benign tumors (n ​= ​5). Preoperative blood samples were collected to assess baseline coagulation parameters, including hematocrit (Hct), international normalized ratio (INR), Quick value, and activated partial thromboplastin time (aPTT). In addition, data on age, sex, anticoagulant medication, and history of venous thromboembolism (VTE) were recorded. Detailed clinicopathological characteristics of the patient cohort are summarized in Table 1.

**Table 1:** Clinicopathologic data of the brain tumor cohort.

Brain tumor type	Glioblastoma n ​= ​10	Brain metastasis^a^ n ​= ​10	Low grade^b^ n ​= ​2	Benign^c^ n ​= ​5
**Age [years]**				
Average	66.1	59.4	63.0	61.4
Median	62.6	57.5	62.9	63.7
Range	(50.8–85.9)	(36.6–75.4)	(58.2–67.8)	(42.0–76.3)
**Gender [n]**				
Female [%]	40.0	20.0	50.0	25.0
Male [%]	60.0	80.0	50.0	75.0
**Baseline coagulation parameters [average]**^d^				
Hct [%]	36.0 ​± ​6.9	38.0 ​± ​5.2	40.0 ​± ​13.0	41.0 ​± ​11.1
INR	1.03 ​± ​0.07	1.00 ​± ​0.15	0.93 ​± ​0.31	0.99 ​± ​0.25
Quick [%]	96.8 ​± ​13.9	98.9 ​± ​15.7	112.0 ​± ​34.4	101.5 ​± ​29.0
aPTT [sec]	27.4 ​± ​3.3	23.6 ​± ​8.7	27.1 ​± ​7.6	30.5 ​± ​8.8
**Anticoagulative treatment [%]**	20.0	30.0	0.0	40.0
**Prior VTE [%]**	0.0	10.0	0.0	0.0

a NSCLC, melanoma, gastric cancer, uterine carcinoma, parotid carcinoma.

b Ependymoma, oligodendroglioma.

c Meningioma and neurinoma.

d Including direct oral anticoagulants (Apixaban, Clexane) and platelet aggregation inhibitor (ASS).

### TTFields exposure

The inovitro™ system (Optune-Gio, Novocure LTD; Haifa, Israel) was used to generate the TTFields. Fresh blood samples were taken from healthy donors (n ​= ​7) and patients (glioblastoma: n ​= ​10; brain metastases n ​= ​11; benign tumors: n ​= ​5; low-grade tumors: n ​= ​2). Tumor cells were harvested, counted, and 3 ​× ​10^4^ ​cells were seeded into chamber slides, followed by overnight incubation at 37 ​°C in a humidified atmosphere of 5 ​% CO_2_ to allow proper cell attachment before treatment. Short-term TTFields treatment was done as follows: blood samples or tumor cells were exposed to 250 ​kHz for 1h or 24 ​h at 20 ​°C in a humidified atmosphere of 5 ​% CO_2_ as described before [12,15]. For consistency, the term 'short-term TTFields exposure' will be used throughout the study. This refers to *in vitro* treatment durations of 1 and 24 ​h, selected to investigate the early biological effects in a controlled setting. These durations are not intended to reflect clinical treatment times. Control samples were incubated at 37 ​°C and 5 ​% CO_2_ in an incubator, but without TTFields. Thereafter, multiple analyses either using cells, whole blood or plasma (purified by centrifugation: 2000×*g*, 8min) were done.

### Flow cytometry

After exposure to TTFields, cells were harvested, washed and counted. Approximately 1 ​× ​10^5^ ​cells were resuspended in 200 ​μl staining buffer (PBS, 2 ​mM EDTA, 2 ​% BSA). For staining tissue factor, 1 ​μl of the appropriate monoclonal antibody against CD142 (Biolegend, San Diego, USA) was added to the staining solution and cells were incubated for 20 ​min at room temperature (RT) in the dark. Following this incubation, 200 ​μl of Yo-Pro 1 staining solution (1:5000) was added, and the cells were incubated for an additional 10 ​min at RT. Subsequently, 300 ​μl of phosphate-buffered saline (PBS) were added, and the cells were subjected to a centrifugation step at 500 ​g for 5 ​min. The supernatant was removed and cells were resuspended in 200 ​μl in staining solution. Detection was performed using the FACSVerse™ (BD, Franklin Lakes, USA).

### ClotPro & clinical chemistry analysis

Blood samples and GBM cells were collected and subjected to ClotPro® viscoelastometry for performing extrinsic test (EX-test) and functional fibrinogen test (FIB-test). Coagulation times (CT in sec) were recorded along with assessment of maximum clot firmness (MCF in millimeters). Additionally, we assessed the platelet component of clot formation by calculating the difference between the EX-test and FIB-test.

Additionally, whole blood samples were conducted to clinical chemistry analysis by measuring the following analytes from the resulting plasma: fibrinogen, factor II, factor VIII, and factor XIII. Additionally, quick and D-dimers.

### Isolation of extracellular vesicles, Nanoparticle Tracking Analysis (NTA), and analysis of procoagulant activity

EVs were isolated from ∼0.5 ​ml of plasma stored at −80 ​°C using differential centrifugation. The plasma was diluted (1:2, 1x sterile PBS) and subjected to medium-speed centrifugation (16,000×*g*, 10 ​min, 4 ​°C) to remove cell debris and large particles. The EV-containing supernatant was transferred to an ultracentrifugation tube and filled with 1x sterile PBS. High-speed ultracentrifugation was performed on the Beckmann Coulter Optima XPN-80 ultracentrifuge with a SW40 Ti swinging bucket rotor (120,000×*g*, 120 ​min, 4 ​°C). Pelleted EVs were diluted in 1x sterile PBS and subjected to Nanoparticle Tracking Analysis (NTA) using NanoSight® Ltd. to quantify the size and concentration. Video recordings were made five times for 30 ​s, after which the mean values of the concentration and size of the EV population were calculated using NTA 3.3 software. PBS particles were excluded by measuring 1x sterile PBS and subtracting them from the samples.

Clotting time of normal plasma and EVs was measured using a coagulometer preheated to 37 ​°C. EVs were diluted in PBS to a final concentration of 1 ​× ​10^9^ particles/ml. Normal plasma, EV suspension, and CaCl_2_ were sequentially added, and clotting time was recorded. TF abundance on EVs was quantified using a sandwich ELISA (Elabscience®, Catalog No: E-EL-H0040) with EVs, following the manufacturer's protocol. Optical density was measured at 450 ​nm using a Tecan microplate reader.

### Microarray analysis

For gene expression analysis, GBM cells treated with TTFields or untreated controls were harvested, counted, and stored at −20 ​°C until RNA isolation. Total RNA was extracted, quantified, and subjected to gene expression profiling using Applied Biosystems™ Clariom™ D arrays (formerly Affymetrix, Thermo Fisher Scientific) as previously described [16]. To identify differentially expressed genes (DEGs), datasets were filtered using an adjusted p-value <0.05 and a fold-change (FC) threshold of ±2.

### Functional enrichment analyses

Enrichment analysis was performed using the DEGs identified in the microarray analysis. The web-based tool g:Profiler was used to identify the top enriched pathways. To ensure statistical rigor and minimize false positives, the false discovery rate (FDR) threshold was set to ​< ​0.05.

The analysis included annotations from Gene Ontology (GO), covering Biological Processes, Molecular Function and Cellular Component categories, as well as from the Kyoto Encyclopedia of Genes and Genomes (KEGG).

### Co-culture model of blood and GBM cells

Cells (HROG04, HROG13, GBM06, GBM15) were seeded, and after 24 ​h of attachment, the medium was removed and 2 ​ml of blood from a healthy donor was added. The co-culture was then exposed to TTFields for 24 ​h. After incubation, the blood was collected, and clotting time was measured. Following the measurement, the blood was centrifuged, and the plasma was used for EV isolation. The GBM cells from the co-culture experiment were used for flow cytometry analysis.

### Statistical information

All values are presented as mean ​± ​SD. Statistical evaluation was performed using GraphPad PRISM software, version 10.1.2 (GraphPad Software, San Diego, CA, USA; RRID:SCR_002798). The criterion for significance was set at p ​< ​0.05. After proving the assumption of normality (Shapiro–Wilk test), the unpaired student's t-test was done for two group analysis. If the normality test failed, the Mann-Whitney *U* Test was performed. The following symbol was used: ∗ vs. ctrl.

## Results

### Short-term TTFields exposure significantly alters blood coagulation by prolonging fibrin polymerization

**Fig. 1:**
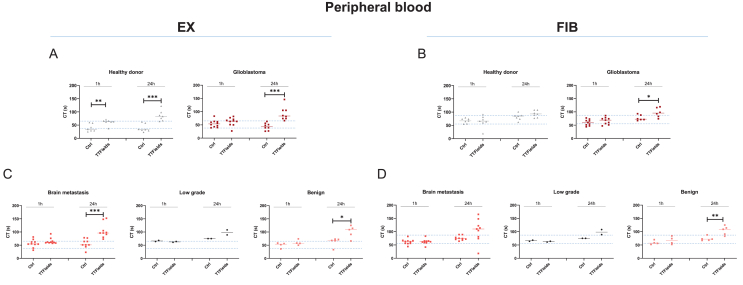
**Clotpro® Hemostasis test reveals TTFields-driven changes in coagulation.** Peripheral blood samples were taken from healthy donors (n ​= ​7) and patients with diverse brain tumor (glioblastoma, n ​= ​10; brain metastasis, n ​= ​10; benign tumors, n ​= ​5); low-grade tumor, n ​= ​2) and exposed to 1 ​h and 24 ​h TTFields, respectively. Thereafter, viscoelastic coagulation times (CT) for (A, C) the extrinsic pathway (EX-test; TF-activated assay) and (B, D) fibrinogen function after platelet inhibition (FIB-test) were measured. Short-term TTFields significantly delayed coagulation times in GBM and brain metastasis patients. Unpaired *t*-test; Individual values are shown, including the median. ∗*p ​<* ​0.05; ∗∗*p ​<* ​0.01; ∗∗∗*p ​<* ​0.001 vs. ctrl. (A–D) Shown are single values ​+ ​median. The blue dotted lines indicate the reference range.

To evaluate the impact of TTFields on coagulation, peripheral blood samples from GBM patients and age- and gender-matched healthy donors (HD) were exposed to 250 ​kHz TTFields for 1 and 24 ​h (Fig. 1). Coagulation dynamics were analyzed using the Clotpro® Hemostasis test, specifically assessing the extrinsic pathway (EX-test; TF-activated assay) and fibrinogen function following platelet inhibition (FIB-test).

Viscoelastic coagulation times (CT), measured via the EX-test, were significantly prolonged in TTFields-treated blood from HDs (24h: p ​< ​0.001 vs. ctrl, Fig. 1A). A similarly prolonged coagulation time was observed in the peripheral blood of GBM patients (24h: p ​< ​0.001). Interestingly, in this patient cohort, but not in HDs, prolonged coagulation times after 24-h TTFields were associated with delayed fibrin polymerization (24h: p ​< ​0.05 vs. ctrl; Fig. 1B).

Then, we analyzed blood samples from patients with brain metastases, low-grade gliomas, and benign tumors (Fig. 1C and D). While TF-driven extrinsic coagulation was prolonged in patients with brain metastases and benign tumors, this effect was only observed after 24 ​h of TTFields. Although fibrin polymerization was similarly delayed, this change did not reach statistical significance. No coagulation alterations were observed in low-grade gliomas. This suggests that prolonged coagulation and delayed fibrin polymerization are specific to high-grade GBM.

### Short-term TTFields exposure reduces clot rigidity in blood of GBM patients

**Fig. 2:**
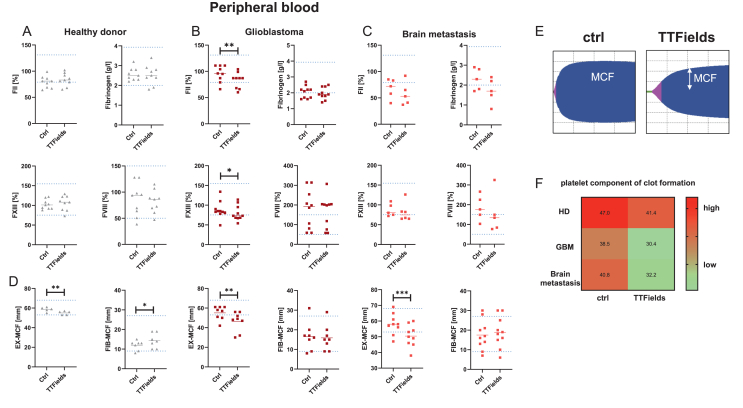
**Clinical chemistry & Clotpro® Hemostasis test identifies specific alteration of the extrinsic pathway after TTFields-exposure.** Peripheral blood samples were taken from healthy donors (n ​= ​5–9) and patients (glioblastoma, n ​= ​8–10; brain metastasis, n ​= ​5–9) and exposed to 24 ​h TTFields. (A, B, C) Markers of coagulation activation, including FII, fibrinogen, FXIII, and FVII were quantified. This analysis identified reduced FII and FXII activity exclusively in GBM patients. (D) Assessment of clot rigidity and stability by measuring maximum clot firmness (MCF) in the EX-test and the FIB-test. Clot rigidity was significantly lower after TTFields exposure, across all groups. Unpaired *t*-test; Individual values are shown, including the median. ∗*p ​<* ​0.05; ∗∗*p ​<* ​0.01; ∗∗∗*p ​<* ​0.001 *vs*. ctrl. (A–D) Shown are single values ​+ ​median. The blue dotted lines indicate the reference range. (E) Exemplary Clotpro® measurement curves from ctrl and TTFields-exposed blood of GBM patients. Plots show altered clot rigidity after short-term TTFields exposure. (F) Analysis of the platelet component within the clots by calculating the difference between EX_MCF_ and FIB_MCF_. In all groups, TTFields exposure reduced the platelet component, especially in GBM patients.

The experiments described above revealed delayed fibrin polymerization, likely influenced by multiple factors, including alterations in fibrinogen quantity or quality, thrombin generation, or FXIII-mediated fibrin cross-linking. Given the potential clinical implications—such as an increased risk of bleeding disorders or thrombosis due to unstable clot formation—we conducted a detailed investigation into the effects of TTFields on local blood coagulation. Blood samples were collected from HDs, GBM patients, and individuals with brain metastases, and key compounds of the plasmatic coagulation system were quantified following 24 ​h TTFields exposure (Fig. 2).

First, by examining samples from HDs, no significant changes in any of the analyzed coagulation markers were seen (Fig. 2A). Analyzing coagulation markers in the plasma of GBM patients (Fig. 2B) revealed that FII (prothrombin) activity, a critical factor in thrombin generation and clot formation, as well as FXIII, which is essential for fibrin clot stabilization, were significantly reduced (*p ​<* ​0.01 and *p ​<* ​0.05 vs. ctrl, Fig. 2B). Fibrinogen levels and FVIII activity, were not altered after TTFields, suggesting intact fibrin production and platelet aggregation in GBM patients’ blood. In blood samples from brain metastasis patients, no alterations were detected and the activity of the analyzed markers remained comparable to untreated controls (Fig. 2C). Other hemostastic parameters, such as quick and D-dimer levels, remained unchanged after TTFields across all samples (Supplementary Fig. 1).

To further assess the impact of TTFields on clot rigidity and stability, we measured maximum clot firmness (MCF) across all cohorts (Fig. 2D). We focused on clots formed through the extrinsic coagulation pathway (=EX-MCF) and clots primarily dependent on fibrinogen function (=FIB-MCF). TTFields significantly reduced EX-MCF in all samples, leading to the formation of weaker and less stable clots formed through the extrinsic coagulation pathway (exemplary plots are shown in Fig. 2E). However, fibrinogen function remained largely unaffected, as FIB-MCF was altered only in HDs but not in GBM or brain metastasis patients (Fig. 2D). In GBM patients', but not in HDs’ blood, the reduction in clot firmness was primarily attributed to **decreased platelet count**, as indicated by the difference between EX_MCF_ and FIB_MCF_ (Fig. 2F), suggesting that clot stability is predominantly fibrinogen-dependent.

In summary, TTFields exposure interferes with blood coagulation in GBM patients leading to localized prolonged coagulation times and the formation of less stable clots, while overall hemostasis remains preserved.

### Short-term TTFields exposure reduces the procoagulant activity of extracellular vesicle secretion

**Fig. 3:**
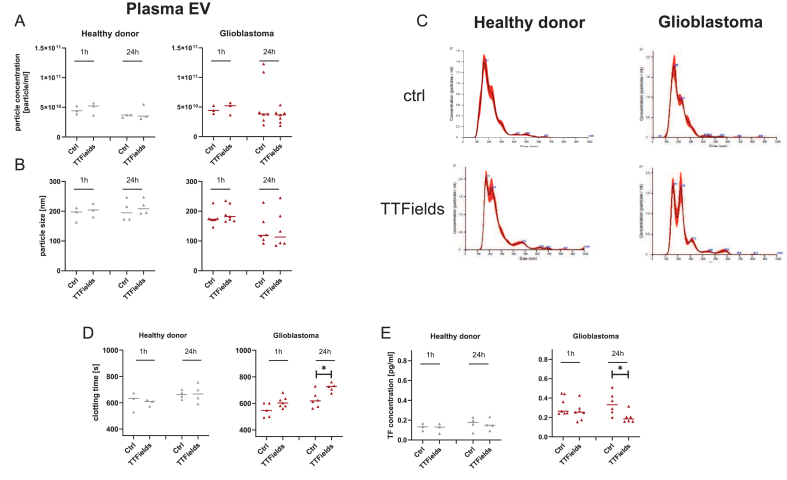
**TTFields alter blood EV secretion and procoagulant activity.** EVs were isolated from plasma samples of healthy donors (n ​= ​3) and GBM patients (n ​= ​7), following 1h and 24 ​h TTFields exposure, respectively. (A, B) EVs were purified by differential ultracentrifugation, and their (A) concentration and (B) mean size were measured via NTA analysis. Coagulation time in normal plasma was assessed as described in material and methods. While TTFields exposure did not alter the total EV quantity, it significantly reduced procoagulant activity in GBM patients but not in healthy donors (HD). (C) Exemplary histograms showing the size distribution of EVs. Unpaired *t*-test; Individual values are shown, including the median. ∗*p ​<* ​0.05 *vs*. ctrl. (D) Clotting time of EVs was measured using a coagulometer preheated to 37 ​°C. EVs were diluted in PBS to a final concentration of 1 ​× ​10^9^ particles/ml and clotting time was recorded. ∗*p ​<* ​0.05 *vs*. ctrl. (E) TF abundance on EVs was quantified using a sandwich ELISA with EVs (corresponding to 1 ​× ​10^9^ particles), following the manufacturer's protocol. ∗*p ​<* ​0.05 *vs*. ctrl. (A, B, D, E) Shown are single values ​+ ​median.

Extracellular vesicles (EVs) play a critical role in regulating and amplifying coagulation. Excessive EV secretion is linked to heightened procoagulant activity and may serve as potential biomarkers. Here, we focused on blood-derived EVs from HDs and GBM patients, as TTFields significantly affected plasmatic coagulation in the latter cohort (Fig. 3).

EV quantification revealed no statistically significant changes following 1 and 24 ​h of TTFields exposure (Fig. 3A). The mean EV size remained consistent between groups, ranging from 130 to 200 ​nm (Fig. 3B). However, GBM-derived EVs—regardless of treatment—were generally smaller than those from HDs (24h: *p* ​= ​0.05). Classical clotting assays using pooled normal plasma identified that EVs from GBM patients exhibited significantly prolonged clotting times after 24 ​h of TTFields exposure (*p ​<* ​0.05 vs. control, Fig. 3D and E), accompanied by a significant reduction in TF levels (*p ​<* ​0.05 vs. control). In contrast, EVs from HDs showed no difference in clotting time or TF concentration, providing additional evidence for a specific effect of TTFields on cellular and subcellular events particularly relevant in GBM.

### Short-term TTFields exposure alters *F3* and *ITGA2* expression on patient-derived GBM cells

**Fig. 4:**
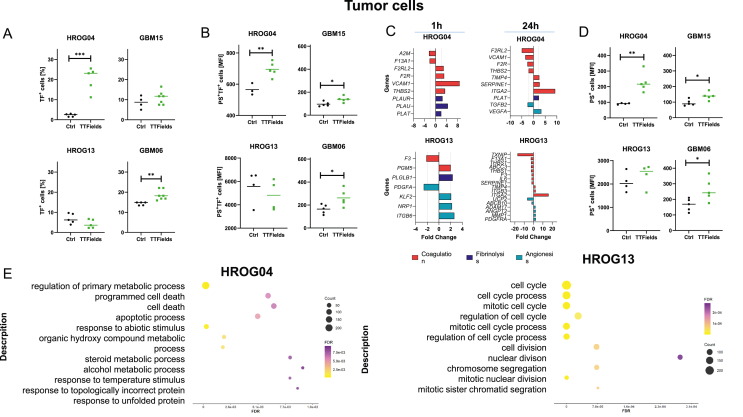
**TTFields exposure of GBM cells reveals phosphatidylserine-mediated decoding of tissue factor and triggers cellular stress responses.** Patient-derived cell lines (n ​= ​4; two with unmethylated MGMT promoter status: HROG04, GBM15 and two with methylated MGMT: HROG13, GBM06) were exposed to short-term TTFields exposure or control conditions for 24 ​h. Thereafter, read-out was done. (A) Quantification of TF abundance on GBM cells (in %) and (B) analysis of PS-mediated TF decoding (MFI), measured via flow cytometry. Unpaired *t*-test; Individual values are shown, including the median. n ​= ​4–6 biological replicates; ∗*p ​<* ​0.05 *vs*. ctrl. (A, B) (A–D) Shown are single values ​+ ​median. (C) Gene expression analysis using Applied Biosystems™ Clariom™ D arrays was done after 1h and 24h TTFields exposure, respectively. Differentially expressed genes (DEGs) were identified by filtering datasets using an adjusted p-value <0.05 and a fold-change (FC) threshold of ±2. Genes involved in coagulation, fibrinolysis, and angiogenesis were altered after TTFields exposure, with cell line-specific differences seen for individual gene sets. (D) Flow cytometric quantification of PS (MFI), indicative of apoptosis. Unpaired *t*-test; Individual values are shown, including the median; ∗*p ​<* ​0.05, ∗∗p ​< ​0.01 *vs*. ctrl. Shown are single values ​+ ​median. (E) GO enrichment analysis was done from gene expression analysis using Applied Biosystems™ Clariom™ D arrays and based on differentially expressed genes, identified by filtering datasets using an adjusted p-value <0.05 and a fold-change (FC) threshold of ±2. Genes involved in different cellular stress responses were included, identifying the successful alteration of cellular metabolism by TTFields.

TF is frequently expressed on the tumor cells’ surface and enhances the hypercoagulable phenotype. To assess the impact of TTFields on the procoagulant activity of tumor cells, we quantified surface TF levels and conducted transcriptomic analysis on four patient-derived GBM cell lines: two with unmethylated MGMT promoter status (HROG04, GBM15) and two with methylated MGMT (HROG13, GBM06) (Fig. 4).

Our results showed increased TF abundance after TTFields exposure, reaching statistical significance in HROG04 and GBM06 (Fig. 4A, Supplementary Fig. 2A). In contrast, TF-positive cells were reduced in HROG13-MGMT^meth^ (vs. ctrl). Mechanistically, TF was translocated to the cell surface in association with phosphatidylserine (PS) (Fig. 4B). The mean fluorescence intensity (MFI) of TF^+^/PS^+^ cells reflected TF expression levels. Specifically, HROG04, GBM15, and GBM06 ​cells showed a significantly higher MFI of TF^+^/PS^+^ cells (*p ​<* ​0.01 and *p ​<* ​0.05 vs. ctrl.), whereas in HROG13, the MFI decreased.

Transcriptomic analysis identified key genes involved in tumor-driven coagulation fibrinolysis, and angiogenesis (Fig. 4C). In HROG04 ​cells, coagulation-associated genes *A2M* and *F13A1* were downregulated after 1 ​h, while vascular homeostasis-associated genes *VCAM1 (*Vascular Cell Adhesion Molecule 1) and *THBS2 (thrombospondin-2)*, together with members of the protease-activated receptor (PAR) family, i.e. *F2R (*coagulation factor II receptor) and *F2RL2 (*Coagulation Factor II Receptor-Like 2), were upregulated. Plasminogen activator genes (*PLAU, PLAUR, PLAT*), which promote fibrinolysis, also showed increased expression.

After 24 ​h, similar trends persisted, with additional upregulation of platelet adhesion-related genes *ITGA2* and *SERPINE1*, both involved in normal blood clotting. Angiogenesis-related genes showed a divergent profile, with some being upregulated (e.g. *VEGFA, TIMP4*) and others being downregulated (e.g. *VCAM1*).

In HROG13 ​cells, *F3* (encoding for TF) remained below control values, after 1 and 24 ​h TTFields (Fig. 4C), consistent with reduced TF abundance at the protein level. *PGM5*, an indirect regulator of GBM cell–coagulation interactions, was upregulated after 1 ​h. Angiogenesis-associated genes, primarily involved in new blood vessel formation (e.g. *KLF2, NRP1,* and *ITGB6*), showed transient upregulation at 1 ​h but returned to baseline by 24 ​h. *TXNIP*, the key regulator of oxidative stress, was downregulated at 24 ​h, while *ITGA2*, similar to that seen in HROG04 ​cells, was highly upregulated, implicating a global role of this gene in response to TTFields. Comparable effects on angiogenesis-related genes were observed.

Given *ITGA2*'s consistent deregulation across both cell lines—implicating roles in coagulation, invasion, and migration—we analyzed its expression after 72 ​h TTFields. *ITGA2* remained elevated in HROG04 but returned to baseline in HROG13 (Supplementary Fig. 2B).

These findings confirm that TTFields interfere with key mechanisms of hypercoagulation in GBM, modulating TF expression, coagulation pathways, and genes involved in fibrinolysis and angiogenesis.

### Short-term TTFields exposure triggers complex stress responses in patient-derived GBM cells

TTFields may interfere with tumor-driven coagulation via PS-mediated TF decoding and gene modulation. Additionally, tumor cells exhibited significantly higher levels of PS (Fig. 4D), independent of TF expression. As this is indicative of apoptosis, we examined transcriptomic changes related to cell growth and cell death (Fig. 4E, Supplementary Fig. 3).

GO enrichment analysis in HROG04 ​cells revealed that 1 ​h TTFields exposure led to an enrichment of pathways involving the binding of molecules, including ions, proteins, and nucleic acids (Supplementary Fig. 3). A similar enrichment pattern was observed after 24 ​h (Fig. 4E). Anabolic processes, such as the regulation of macromolecule biosynthesis and protein metabolism, related to cell development and cell cycle, were enriched after 1 ​h of TTFields. After 24 ​h, a shift toward apoptotic processes and the unfolded protein response was detected that likely contributed to programmed cell death.

### Co-culture of blood and tumor cells confirms interference of TTFields with coagulation

**Fig. 5:**
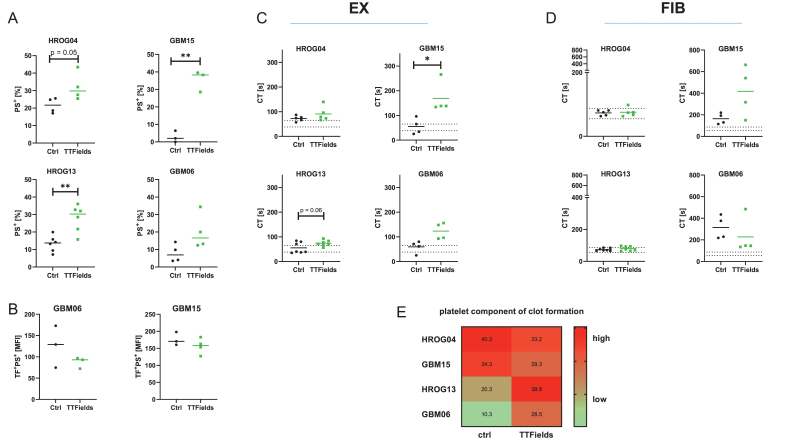
**Allogeneic co-culture of GBM cells and peripheral blood confirms the coagulation-immune regulation axis.** Blood from healthy donors was added to GBM cells and exposed to 24 ​h TTFields. (A) Flow cytometric quantification of PS (MFI) on GBM cells, indicative of tumor cell apoptosis in this co-culture model. (B) Co-staining for PS and TF confirmed that the increased PS exposure resulted from apoptosis induction rather than TF decoding. N ​= ​3–6 biological replicates; unpaired *t*-test; Individual values are shown, including the median; ∗∗*p ​<* ​0.01 *vs*. ctrl. Viscoelastic coagulation times (CT) in the blood for (C) the extrinsic pathway (EX-test) and (D) fibrinogen function after platelet inhibition (FIB-test). This analysis confirmed the delayed coagulation time after TTFields exposure. Unpaired *t*-test; Individual values are shown, including the median. ∗*p ​<* ​0.05 vs. ctrl. The blue dotted lines indicate the reference range. (A–D) Shown are single values ​+ ​median. (E) Analysis of the platelet component within the clots by calculating the difference between EX_MCF_ and FIB_MCF_. This analysis yielded cell line-specific results, with lower platelet counts after co-culture with MGMT^unmeth^ cell lines (HROG04, GBM15) and higher counts after co-culture with MGMT^meth^ cell lines (HROG13, GBM06).

Finally, a tumor-blood co-culture model was employed to investigate the impact of TTFields on coagulation in a complex *in vitro* system (Fig. 5). HD blood was added to GBM cells and exposed to TTFields for 24 ​h. In this co-culture model, the antitumor effects of TTFields were maintained, as evidenced by an increase in apoptotic cells across all patient-derived GBM cell lines (HROG04, GBM15, HROG13, and GBM06), with statistical significance observed in GBM15 and HROG13 (*p ​<* ​0.01 *vs*. ctrl, Fig. 5A). Co-staining for PS and TF confirmed increased PS exposure due to apoptosis rather than TF decoding. In GBM06 and GBM15, the MFI of TF^+^/PS^+^ cells was lower after TTFields exposure (Fig. 5B).

In blood samples, clotting time, measured *via* the EX-test, was likewise prolonged in TTFields-treated samples (GBM15: *p ​<* ​0.05 vs. ctrl, Fig. 5C). This prolonged coagulation again correlated with delayed fibrin polymerization (Fig. 5D). However, clot firmness remained unaffected (Supplementary Fig. 2), and **platelet count was only reduced in samples co-cultured with the MGMT^unmeth^-cell lines (HROG04, GBM15)**, as indicated by the difference between EX_MCF_ and FIB_MCF._ Importantly, platelet function itself was not impaired (Fig. 5E). Additional clot firmness values for all co-culture conditions are presented in Supplementary Fig. 4.

## Discussion

Through a preclinical *ex vivo* study, we provide compelling evidence that TTFields interfere with coagulation, primarily affecting the extrinsic, TF-driven coagulation pathway. The absence of reported systemic bleeding complications in published TTFields studies supports the hypothesis that associated thrombotic phenomena are likely driven by localized pathophysiological interactions between individual cells rather than generalized hemostatic imbalance [11]. Prolonged blood coagulation times were observed across different patient cohorts, irrespective of the underlying malignancy. In GBM patients, this was associated with delayed fibrin polymerization and the formation of a less stable fibrin network, likely composed of thinner, more branched fibers [17], with reduced stiffness and increased plasticity. The reduced clot stiffness after TTFields exposure was attributed to lower FII (prothrombin) and FXIII activity. FXIII, activated by thrombin to form FXIIIa, cross-links fibrin strands, enhancing the physical durability of the clot and protecting it from fibrinolysis [18]. Fibrinogen levels were not significantly affected by TTFields, suggesting that clot structure rather than normal hemostatic activity was modulated. Consistently, fibrin production and platelet aggregation remained comparable to pre-TTFields levels. Although platelets are a-nucleated and do not undergo cell division, a reduction was observed across different patient cohorts and healthy donors, with most pronounced impact in GBM patients. Our data therefore imply that TTFields primarily influence the final stages of coagulation, particularly fibrin polymerization. The exact mechanism remains elusive, as delayed fibrin polymerization in our study was also observed in patients with benign tumors. Although these patients, especially those with advanced age, high body mass index, or a history of thrombosis, may have an elevated thrombosis risk, their overall risk remains below that of GBM patients [19,20]. TTFields may therefore interfere with coagulation factors common to both benign tumors and high-grade GBM. Supporting this, TTFields reduced shedding of procoagulant plasma-derived EVs, an effect specific to GBM patients and absent in healthy controls. Since total EV levels remained unchanged, we propose a direct interference with GBM-associated coagulation factors – underscored by the fact that EV-specific TF levels were reduced. Collectively, these findings in whole blood, plasma, and plasma-derived EVs indicate that TTFields significantly disrupt the extrinsic pathway of plasmatic coagulation, while the intrinsic pathway plays a minor role. While these findings may raise concerns regarding increased bleeding risk, it is important to highlight that no serious hematological toxicities have been reported in clinical studies to date [11]: In the EF-14 ​phase III trial, a large-multicenter study with ∼700 patients, no increased rates of intracranial hemorrhage or other bleeding complications was reported. Also, the incidence of serious adverse events, including pulmonary embolism, was comparable between the TTFields and control groups [11]. Moreover, large post-marketing safety analyses involving over 25,000 TTFields-treated patients with CNS malignancies did not specifically report on thromboembolic outcomes [21]. This supports the hypothesis that the observed changes in clot stiffness and TF/PS expression are likely confined to the tumor microenvironment and do not reflect systemic hemostatic disruption. The localized, non-ionizing nature of TTFields therapy appears to preserve vascular integrity and systemic coagulation homeostasis, consistent with the absence of reported bleeding events in clinical cohorts.

Tumor cells serve as binding surfaces for various coagulation cascade proteins by exposing TF and PS on their outer membrane. The negatively charged PS surface facilitates the assembly of catalytically active coagulation complexes. In GBM, TF is frequently upregulated due to oncogenic events such as EGFRvIII activation and loss of tumor suppressor genes *TP53* and *PTEN* [22]*.* All GBM cell lines used here harbor *TP53* mutations, and exhibit TF expression, with variations ranging from 5 to 15 ​%. TTFields induced TF upregulation on GBM cells, coinciding with PS translocation. PS-mediated TF decoding enables the formation of both the extrinsic and intrinsic tenase complexes, as well as the prothrombinase complex, thereby influencing coagulation dynamics [22]. Although FII levels were significantly reduced in the blood, its receptor F2R and the ligand F2RL were higher on HROG04 ​cells after TTFields, suggesting a compensatory regulatory response. TTFields also modulated the hypercoagulable phenotype of GBM cells by suppressing *A2M* expression and upregulating plasminogen activator genes, both of which influence coagulation and fibrinolysis but may also facilitate tumor cell migration. In HROG13 ​cells, TTFields reduced the hypercoagulable state by modulating *F3* and *TXNIP* expression. The downregulation of *F3* correlated with decreased translocation of PS-exposed TF to the cell surface. *TXNIP* promotes oxidative stress, platelet activation, and aggregation through reactive oxygen species (ROS)-mediated pathways, enhancing procoagulant and inflammatory signaling. The concurrent downregulation of *F3* and *TXNIP* following TTFields exposure suggests an anti-inflammatory effect that ultimately reduces the cells procoagulant phenotype.

In contrast to radiation therapy, which has been shown to upregulate tissue factor expression and promote fibrin accumulation in glioblastoma tissue [23], TTFields did not induce a generalized procoagulant phenotype. Rather, TF expression was reduced in certain GBM models (e.g., HROG13), and EV-associated TF levels decreased consistently. These effects occurred in the absence of overt cytotoxicity and suggest a fundamentally different mechanism of action compared to ionizing radiation, which primarily induces DNA double-strand breaks. Our findings therefore support the notion that TTFields exert local anticoagulant effects not through induction of cell death, but via modulation of coagulation-related signaling pathways.

Furthermore, platelet adhesion-related gene *ITGA2* was significantly upregulated in both cell lines. *ITGA2* is particularly intriguing, as it not only plays a role in normal blood clotting but is also implicated in cell-matrix interactions, immune modulation, and cellular signaling pathways. *ITGA2* is enriched in glioma stem-like cells to promote invasion by modulating PD-L1 and activating STAT3 phosphorylation, enhancing epithelial-mesenchymal transition [[24], [25], [26]]. *ITGA2* upregulation following short-term TTFields returned to baseline levels over time, suggesting an acute stress response rather than sustained stemness enrichment. In support of this, the accumulation of stem-like cells after TTFields is rarely described in the literature. One study reported that stem-like cells are equivalent TTFields sensitivity between stem-like cells and their differentiated counterparts [27], while a recent report identified the EP3–ZNF488 axis as a potential TTFields resistance mechanism, enhancing self-renewal and regulatory network remodeling [28]. Interestingly, both EP3 and ZNF488 are involved in CNS development, platelet aggregation, and inflammation, underscoring the complexity of TTFields sensitivity and resistance mechanisms [[29], [30], [31]]. By identifying TTFields-mediated modulation of tumor and blood coagulation, we propose a novel mechanistic axis linking inflammation and coagulation.

TSP-1 (gene name: *THBS1*) is a matricellular glycoprotein involved in hemostasis, extracellular matrix remodeling, and tumor progression [32]. In GBM, expression of *THBS1* is transcriptionally regulated via the TGF-β/SMAD3 axis and enriched at the invasive tumor margins. It promotes tumor cell migration and invasion by interacting with CD47, and its silencing inhibits glioma growth and enhances sensitivity to anti-angiogenic therapy. Although *THBS1* is often described as anti-angiogenic through CD36-mediated signaling, it can also support pro-tumoral processes such as immune evasion, extracellular vesicle signaling, and resistance to genotoxic stress [[33], [34], [35]]. The observed decrease in *THBS1* expression following TTFields exposure may therefore reflect a complex regulatory shift that not only affects clot architecture, but also modulates tumor–stroma communication and stress adaptation. Importantly, this effect coincides with alterations in platelet function and extracellular matrix signaling, suggesting a broader role of TTFields in reshaping the GBM microenvironment beyond coagulation alone.

A recent study suggested that hypercoagulability in GBM patients is primarily a local phenomenon, with no observed association between circulating tumor cells and thromboembolic events [36]. Hence, our findings are particularly relevant, as we only had access to peripheral blood from GBM patients yet observed significant interference with the extrinsic, TF-driven coagulation pathway. Investigating the impact of **TTFields** on the cerebral circulatory system in clinical settings would be highly valuable.

Thus, no definitive conclusions can yet be drawn regarding a potential protective effect of TTFields on thrombotic risk. Nevertheless, the consistent absence of increased thromboembolic events in clinical trials and real-world data supports the overall hemostatic safety of TTFields.

This study has several limitations: First, patient samples were collected from a single center; limiting generalizability; a multi-center study would be preferable. Second, blood coagulation *ex vivo* was studied in a static system rather than in patients actively receiving TTFields therapy. A longitudinal evaluation of specific coagulation parameters would provide further insight, particularly regarding platelet count and function. Additionally, we focused solely on the short-term effects of TTFields, leaving the long-term impact on local blood coagulation unknown. Thus, how TTFields influence coagulation and affect local inflammatory processes over extended periods remains an open question. Another limitation is the use of an allogenic co-culture system rather than an autologous one. While acquiring both tumor tissue and sufficient patient blood is challenging, the implementation of sophisticated organ-on-chip *ex vivo* models that better mimic microvascular networks *in vitro* could address this issue. Moreover, while our data suggest localized rather than systemic effects, clinical trials are required to assess whether these changes translate into measurable differences in thrombotic or bleeding complications. These limitations should be considered when interpreting the translational relevance of our observations.

Taken together, this study provides the first direct evidence that TTFields modulate coagulation by dysregulating TF activity, suppressing prothrombotic factors, and reprogramming thrombotic gene networks. Clinicians should remain vigilant in monitoring coagulation parameters in GBM patients undergoing TTFields therapy to optimize anticoagulation strategies while minimizing bleeding and thromboembolic complications.

## Data availability statement

All data are available from the corresponding author upon reasonable request.

## Declaration of generative AI and AI-assisted technologies in the writing process

During the preparation of this work the author(s) used ChatGPT and DeepL Write in order to improve the clarity, structure, and linguistic quality of the manuscript, as well as to assist with translation and abstract formatting according to journal requirements. After using these tools, the author(s) reviewed and edited the content as needed and take(s) full responsibility for the content of the publication.

## Funding information

This work was supported by 10.13039/100012634Novocure, which provided the Inovitro™ Device and financial assistance for the procurement of experimental disposables. The funding source had no involvement in the study design, data collection, analysis, interpretation, or preparation of the manuscript.

## Authors' contributions

D.D. conceived and designed the study. A.H., S.S., and A.W. performed the experiments and contributed to sample processing and laboratory work. C.M., T.F., and S.S. analyzed the data and prepared the figures. C.M. and T.F. wrote the main manuscript text, performed bioinformatics analyses, and contributed to data interpretation. D.K. assisted with experimental procedures, data collection, and microarray analysis. T.M.F., D.D., F.G., S.Y.W., P.V., and J.B. provided critical revision of the manuscript. D.D. and F.G. supervised the study and provided conceptual guidance. All authors reviewed and approved the final manuscript.

## Declaration of competing interest

The authors report no personal or financial conflicts of interest. Novocure Ltd. (Haifa, Israel) provided the TTFields device and €5000 in institutional research support related to this work.

## Appendix A Supplementary data

Supplementary Fig. 1 Clinical chemistry for analysis of coagulation activation. Clinical laboratory analysis for quick (%) and d-dimers (mg/l) in blood of healthy donors, GBM and brain metastasis patients showed no difference between TTFields-exposed and control samples. (A-D) Shown are single values ​+ ​median. The blue dotted lines indicate the reference range. Supplementary Fig. 1

Supplementary Fig. 2 Flow cytometric gating strategy & Microarray analysis. (A) Gating strategy for detection of TF and PS on GBM cells. (B) Gene expression analysis using Applied Biosystems™ Clariom™ D array was done after 72h TTFields exposure. Differentially expressed genes (DEGs) were identified by filtering datasets using an adjusted p-value <0.05 and a fold-change (FC) threshold of ±2. Here, expression level of *ITGA2* were determined in HROG04 and HROG13 ​cells, to confirm the induction of an acute stress response. Supplementary Fig. 2

Supplementary Fig. 3 Microarray analysis. Patient-derived cell lines (HROG04, HROG13) were exposed to short-term TTFields exposure or control conditions for 1 ​h. GO enrichment analysis was done from gene expression analysis using Applied Biosystems™ Clariom™ D arrays and based on differentially expressed genes, identified by filtering datasets using an adjusted p-value <0.05 and a fold-change (FC) threshold of ±2. Genes involved in different cellular stress responses were included, identifying the successful alteration of cellular metabolism by short-term TTFields. Supplementary Fig. 3

Supplementary Fig. 4 Allogeneic co-culture of GBM cells and peripheral blood. Blood from healthy donors was added to GBM cells and exposed to 24 ​h TTFields. Assessment of clot rigidity and stability by measuring maximum clot firmness (MCF) in the EX-test and the FIB-test. Clot rigidity was not significantly impaired after TTFields exposure, with only minor cell line-specific differences. unpaired *t*-test; Individual values are shown, including the median; Viscoelastic coagulation times (CT) in the blood for (A) the extrinsic pathway (EX-test; TF-activated assay) and (B) fibrinogen function after platelet inhibition (FIB-test). (A, B) Shown are single values ​+ ​median. The blue dotted lines indicate the reference range. Supplementary Fig. 4
