# Supplementary figures and images for: Tumor treating fields alter local coagulation dynamics in glioblastoma patients
Source: Neurotherapeutics. 2025 Aug 29;22(6):e00715. doi: 10.1016/j.neurot.2025.e00715 (PMC12664522; doi:10.1016/j.neurot.2025.e00715)

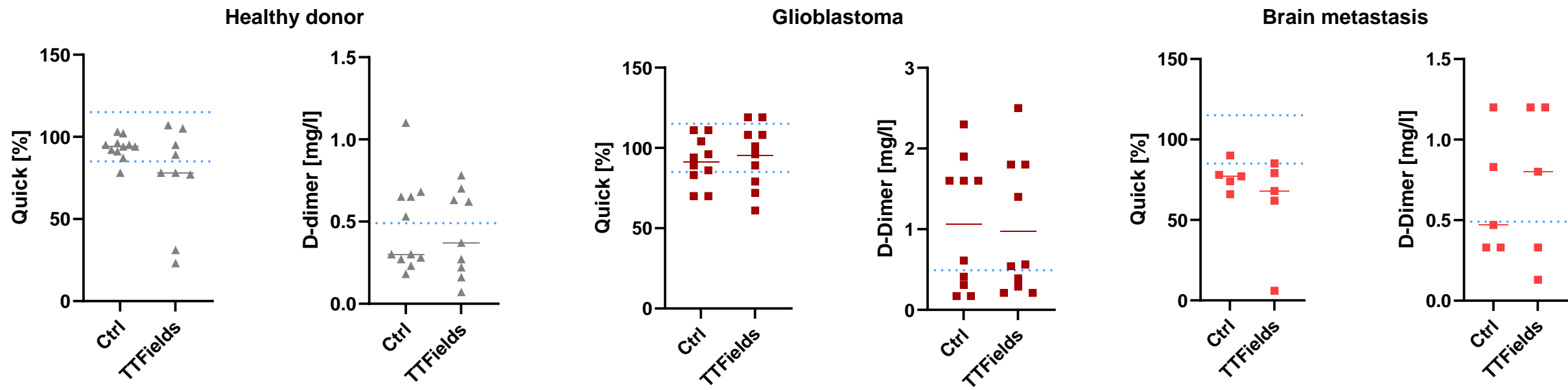

Supplementary Figure 1

Supplement: Supplementary Fig. 1 — Clinical chemistry for analysis of coagulation activation. Clinical laboratory analysis for quick (%) and d-dimers (mg/l) in blood of healthy donors, GBM and brain metastasis patients showed no difference between TTFields-exposed and control samples. (A-D) Shown are single values ​+ ​median. The blue dotted lines indicate the reference range. [file mmc1.pdf]

A

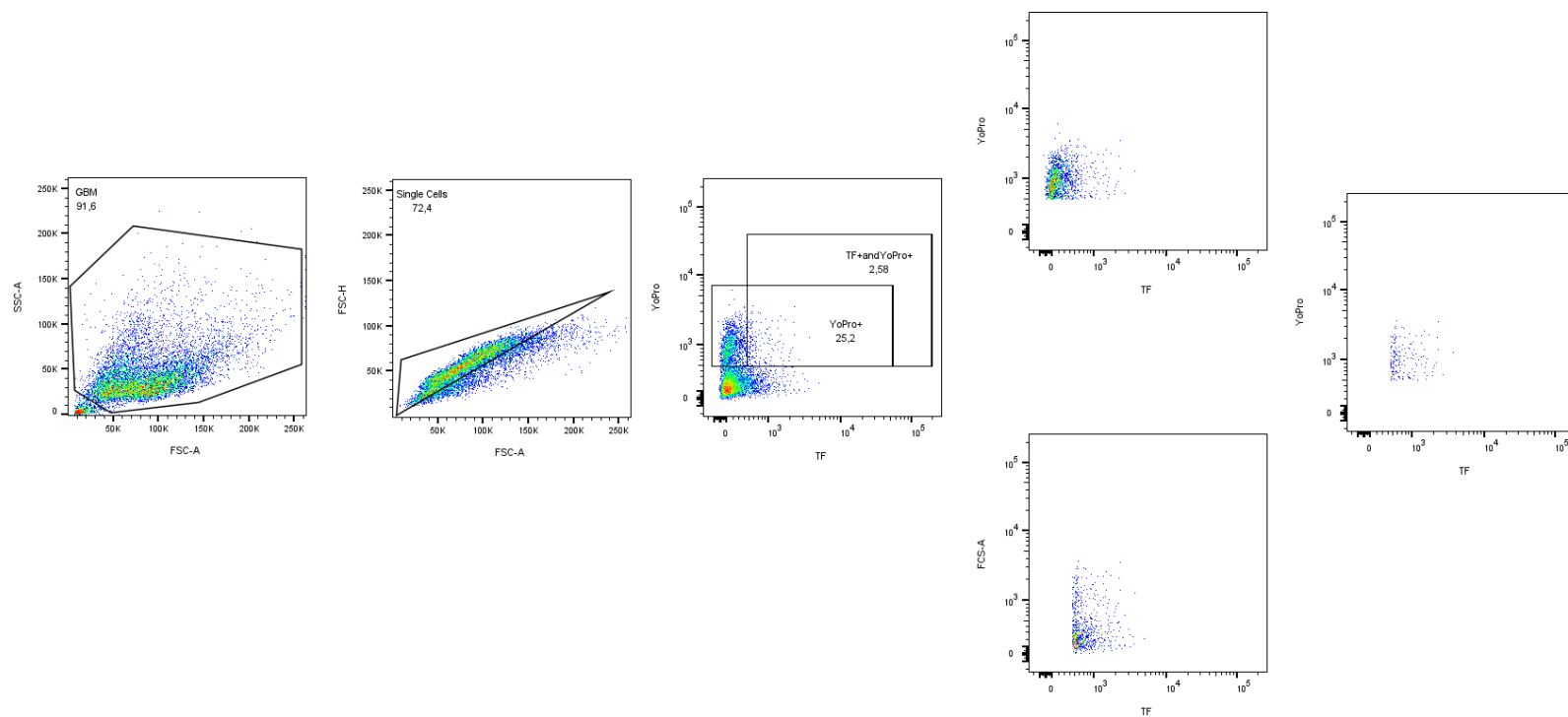

B

72h

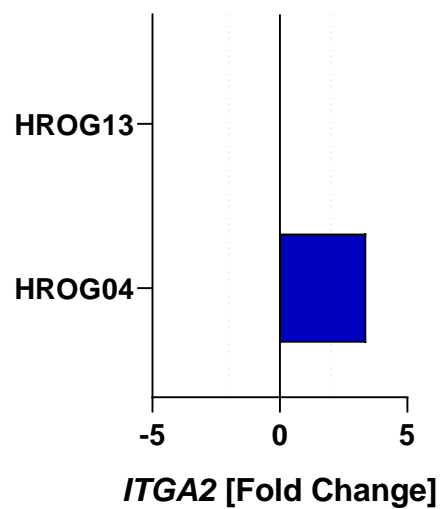

Supplement: Supplementary Fig. 2 — Flow cytometric gating strategy & Microarray analysis. (A) Gating strategy for detection of TF and PS on GBM cells. (B) Gene expression analysis using Applied Biosystems™ Clariom™ D array was done after 72h TTFields exposure. Differentially expressed genes (DEGs) were identified by filtering datasets using an adjusted p-value <0.05 and a fold-change (FC) threshold of ±2. Here, expression level of ITGA2 were determined in HROG04 and HROG13 ​cells, to confirm the induction of an acute stress response. [file mmc2.pdf]

Description

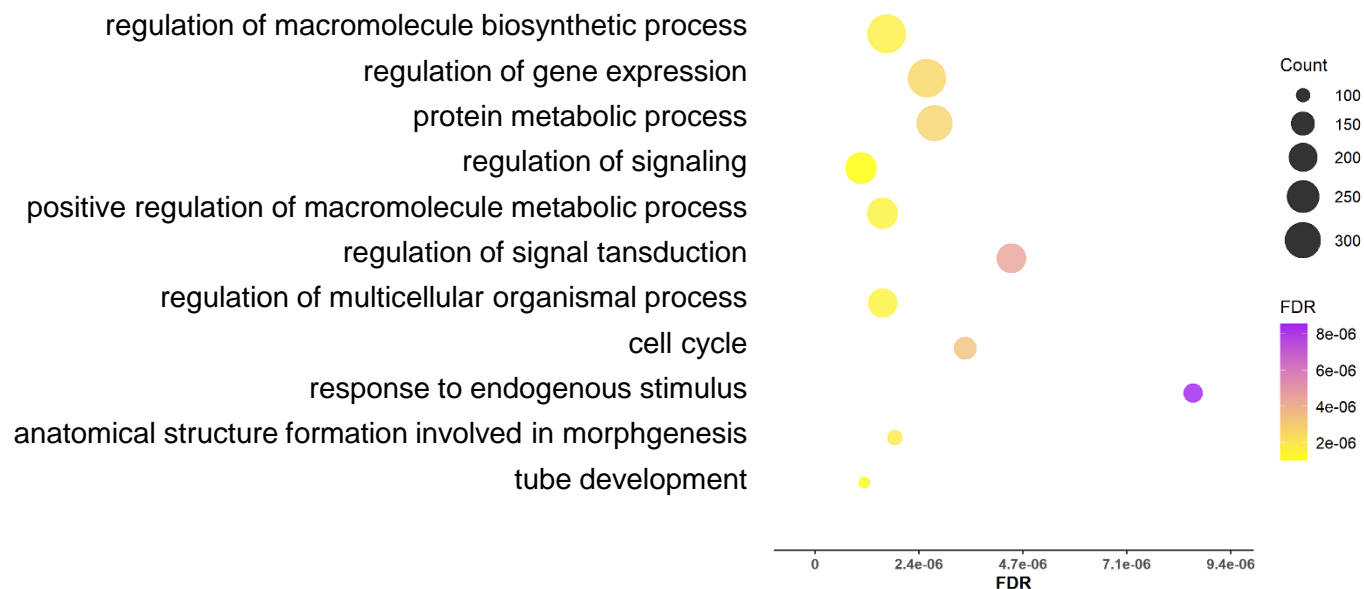

Description

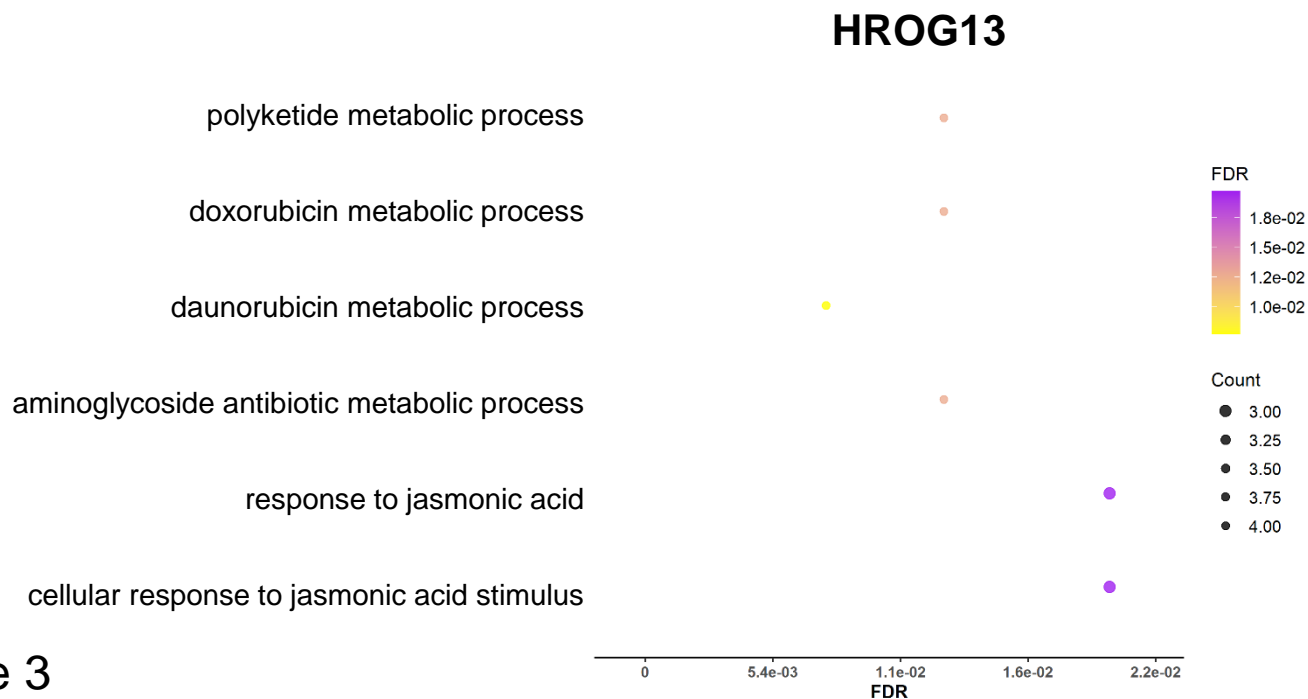

Supplement: Supplementary Fig. 3 — Microarray analysis. Patient-derived cell lines (HROG04, HROG13) were exposed to short-term TTFields exposure or control conditions for 1 ​h. GO enrichment analysis was done from gene expression analysis using Applied Biosystems™ Clariom™ D arrays and based on differentially expressed genes, identified by filtering datasets using an adjusted p-value <0.05 and a fold-change (FC) threshold of ±2. Genes involved in different cellular stress responses were included, identifying the successful alteration of cellular metabolism by short-term TTFields. [file mmc3.pdf]

A

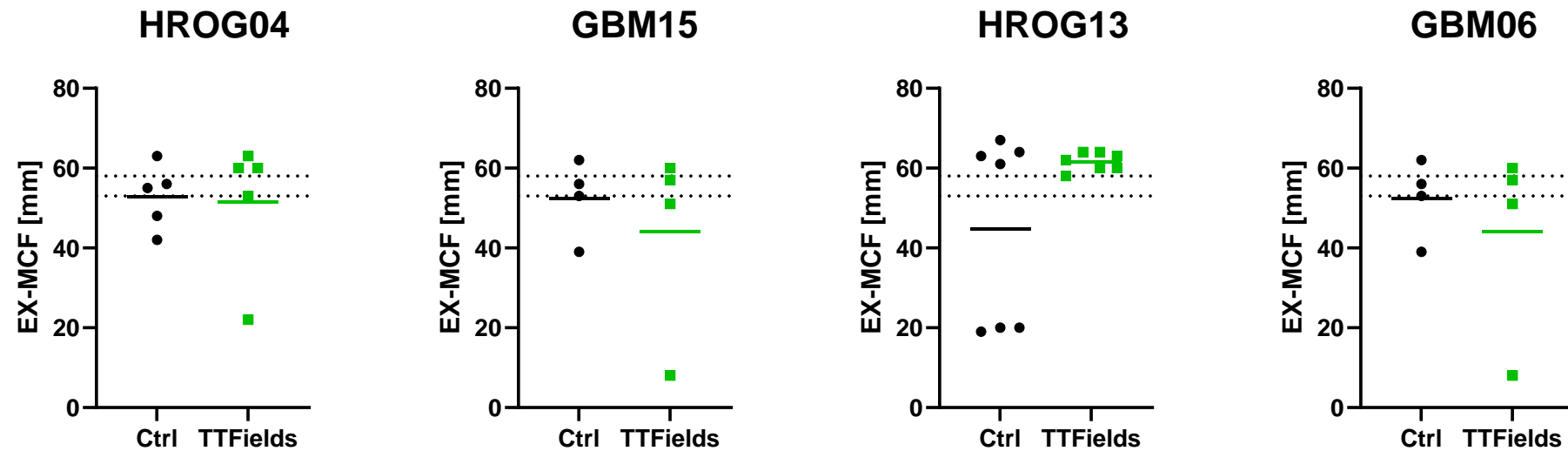

B

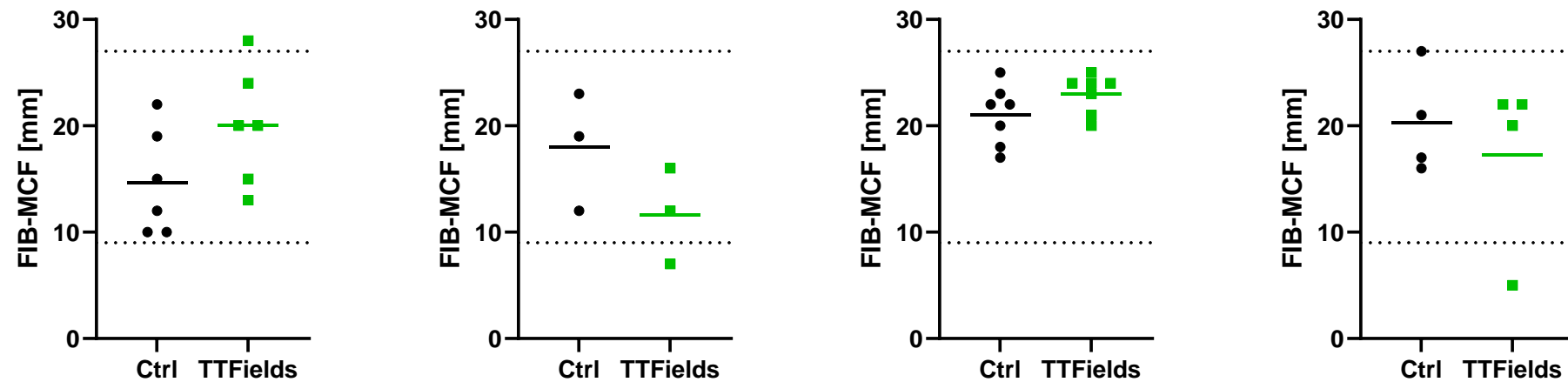

Supplement: Supplementary Fig. 4 — Allogeneic co-culture of GBM cells and peripheral blood. Blood from healthy donors was added to GBM cells and exposed to 24 ​h TTFields. Assessment of clot rigidity and stability by measuring maximum clot firmness (MCF) in the EX-test and the FIB-test. Clot rigidity was not significantly impaired after TTFields exposure, with only minor cell line-specific differences. unpaired t-test; Individual values are shown, including the median; Viscoelastic coagulation times (CT) in the blood for (A) the extrinsic pathway (EX-test; TF-activated assay) and (B) fibrinogen function after platelet inhibition (FIB-test). (A, B) Shown are single values ​+ ​median. The blue dotted lines indicate the reference range. [file mmc4.pdf]
